# Fibro-Sarcoma of the Orbit*Stenographically reported for this journal by C. C. Mapea, Louisville, Ky.

**Published:** 1899-01

**Authors:** J. Morrison Ray

**Affiliations:** Clinical Professor of Ophthalmology, Otology and Laryngology in the University of Louisville, etc., Louisville, Ky.


### FIBRO-SARCOMA OF THE ORBIT.*

BY J. MORRISON RAY, M.D.

Clinical Professor of Ophthalmology, Otology and Laryngology in the University of Louisville, etc., Louisville, Ky.

Two years ago I presented before the Louisville Medico-Chirurgical Society a case of pulsating exophthalmos. To-night I desire to exhibit a case of exophthalmos in which pulsation can not be detected. It is a case of one-sided exophthalmos with some interesting symptoms.

I first saw the patient, Mr. K., on November 20th. He is a laborer, aged thirty years, and has a history of syphilis dating back fifteen years. Two years ago pain began in the left side of his face; it was thought to be neuralgic, probably referred to the teeth. Two teeth were extracted without relief; on the contrary, pain has been more severe since the teeth were drawn. A year ago it was noticed that the left eye bulged slightly, and he began to have blurring of vision in that eye.

In November he presented a dilated fixed pupil; marked exophthalmos; complete ophthalmoplegia; complained of occasional dizzy sensations; suffered pain at the inner corner of the eye, which was always worse at night. Four years ago he was struck quite a severe blow on that side of the head. There is now anesthesia of the eyelid extending some distance on the forehead and on the face below; this was not complete at my first examination. At present ptosis is almost complete. My first examination showed no congestion about the right eye, vision perfect. In the left eye he had vision of 12-200. The ophthalmoscope showed no change in the optic nerve, but there was some venous congestion and obstruction in the left eye.

He had been treated for syphilis ever since appearance of the original sore. The doctor consulted for relief of the neuralgia put him upon iodide of potassium, and every doctor consulted since has administered iodides and mercury without any benefit so far as his eye is concerned.

I have not seen him since last November until to-day. He now complains of intense pain, especially at night, and morphine is required to enable him to get any rest. He also complains of dizziness; his feet "go to sleep;" his hands get numb, etc. His eye is pushed directly forward, there being little or no lateral deviation. His bowels move only once a week unless he takes a purgative. He is now completely blind in the affected eye.

The question arises, what is the cause of this exophthalmos? I examined the antrum by transillumination, but detected no obstruction about either the antrum or ethmoid cells. I believe there is a malignant growth back of the orbit and have proposed surgical interference.

*Stenographically reported for this journal by C. C. Mapea, Louisville, Ky.

REMARKS.

Dr. I. N. Bloom: I do not believe the exophthalmos is due to syphilis.

Dr. A. M. Vance: I saw this man last summer and was given about the history detailed by Dr. Ray. I believe there is a growth back of the orbit, but its origin I do not know. He had been able to work up to the time I saw him, and had been treated for syphilis irregularly since the disease was contracted fifteen years ago. I believe syphilis has something to do with the exophthalmos.

Dr. A. M. Cartledge: It is more than likely the growth is intra-cranial and consequently back of the orbit; penetration into the orbit has occurred, hence the marked bulging of the eye. I can scarcely see how a purely orbital tumor could produce some of the symptoms present.

As to the nature of the growth: We might have a syphilitic deposit in this situation sufficiently organized to resist treatment, especially when we view an organized gumma as a neoplasm, and an organized gumma is about as obstinate to treatment as a sarcoma. It is doing this man an injustice not to make an attempt to relieve him. I do not believe the disease will yield to anti-syphilitic treatment, hence an exploration should be made. I think the tumor will be found combined intra-crani l and orbital, and more than likely it is a sarcoma.

Dr. William Cheatham: I agree with what Dr. Cartledge has said except I believe the growth is purely orbital. I had this man under observation at one time; he was given anti-syphilitic remedies, and the disease progressed notwithstanding the treatment. An operation ought to be performed.

Dr. T. C. Evans: I agree with Dr. Cartledge that the growth involves both the cranium and the orbit. Whether it began in the brain or in the orbit I can not say.

Dr. W. C. Dugan: Whether the growth is cranial or orbital I do not know. I would advise immediate operation.

Dr. J. M. Ray: There are several points which indicate that the growth is orbital and not cranial. First, a tumor growing from the cranial cavity pressing upon the eye would force the eye downward. The eye in this case is forced directly forward. The growth probably springs from the apex of the orbit, and more or less surrounds the eye, thus it follows the apex of the orbit forward. Only one kind of growth of the orbit is likely to do this and that is a tumor of the optic nerve. Growths springing from the orbit, from the ethmoid cells, or the frontal bone, would push the eye aside from its axis. Against the growth springing directly from the apex of the orbit is the fact that the optic nerve shows little change from pressure. Usually where a growth presses upon the nerve some evidence of pressure is visible with the opthalmoscope. On the other hand, a growth starting in the sheath of the optic nerve may push the eye forward without the optic nerve itself being seriously involved.

SUBSEQUENT REPORT.

DR. J. M. RAY: At the last meeting of this society I presented a man with one-sided exophthalmos with a history of gradual protrusion of the eye extending over two years. During the last six months there had been progressive blindness which at the time he was shown had become absolute. Ophthalmoscopic examination showed little evidence of disease of the eye beyond venous engorgement. The eye was pushed forward in line with the axis of the orbit; it was perfectly fixed; there was a dilated pupil; anesthesia of the eyelid and part of the forehead and face, following the distribution of the fifth cranial nerve; with a history of great pain, especially at night, requiring morphine for its relief.

The members who examined the case agreed that there was a growth, the nature of which it was impossible to state, pushing the eye forward. Before presenting the patient I had examined the nose, antrum and frontal sinus by transillumination, finding them perfectly clear, so that the character of the growth was obscure.

A few days later the eye was enucleated at the University Clinic. After removing the eye I explored the orbit and found the whole apex filled with a hard nodular mass. There were three nodules, one filling the outer part of the apex, another coming from the lower portion of the orbit, and still another above. These three masses came together forming a triangle through which passed the optic nerve. There was little or no pressure upon the optic nerve itself. These masses were so closely and firmly attached to the periosteum that they had to be enucleated in small pieces. I removed everything I could from the orbit, leaving the conjunctival and sub-conjunctival tissues to cover the cavity. The optic nerve was resected close to the optic foramen. Nowithstanding this I believe the growth was incompletely removed. In the spheno-maxillary fissure could be felt small nodules which it was absolutely impossible to remove. Specimens were submitted to the pathological laboratory and the pathologist's report reads "Fibrosarcoma involving the periosteum."

The man did very well after the operation. He was kept in the hospital for a week; the discharge of pus was free; there was considerable edema of the lid and tissue overlying the side of the face. Three weeks after removal of the growth there was decided evidence of recurrence. The apex of the orbit now seems to be filled and I think in a short time the growth will entirely fill the orbit. The man has had no pain nor has he taken a dose of morphine since the night after the operation.

